# Forest genomics: Advancing climate adaptation, forest health, productivity, and conservation

**DOI:** 10.1111/eva.12902

**Published:** 2019-12-23

**Authors:** Nathalie Isabel, Jason A. Holliday, Sally N. Aitken

**Affiliations:** ^1^ Laurentian Forestry Centre Canadian Forest Service Natural Resources Canada Québec Canada; ^2^ Canada Research Chair in Forest Genomics Centre for Forest Research and Institute for Systems and Integrative Biology Université Laval Québec Canada; ^3^ Department of Forest Resources and Environmental Conservation Virginia Tech Blacksburg VA USA; ^4^ Centre for Forest Conservation Genetics and Department of Forest and Conservation Sciences University of British Columbia Vancouver Canada

**Keywords:** assisted gene flow, cyberinfrastructure, forest management, genomic selection, hybridization, insect and disease resistance, landscape genomics, nonmodel species, tree breeding

## Abstract

Forest ecosystems provide important ecological services and resources, from habitat for biodiversity to the production of environmentally friendly products, and play a key role in the global carbon cycle. Humanity is counting on forests to sequester and store a substantial portion of the anthropogenic carbon dioxide produced globally. However, the unprecedented rate of climate change, deforestation, and accidental importation of invasive insects and diseases are threatening the health and productivity of forests, and their capacity to provide these services. Knowledge of genetic diversity, local adaptation, and genetic control of key traits is required to predict the adaptive capacity of tree populations, inform forest management and conservation decisions, and improve breeding for productive trees that will withstand the challenges of the 21st century. Genomic approaches have well accelerated the generation of knowledge of the genetic and evolutionary underpinnings of nonmodel tree species, and advanced their applications to address these challenges. This special issue of Evolutionary Applications features 14 papers that demonstrate the value of a wide range of genomic approaches that can be used to better understand the biology of forest trees, including species that are widespread and managed for timber production, and others that are threatened or endangered, or serve important ecological roles. We highlight some of the major advances, ranging from understanding the evolution of genomes since the period when gymnosperms separated from angiosperms 300 million years ago to using genomic selection to accelerate breeding for tree health and productivity. We also discuss some of the challenges and future directions for applying genomic tools to address long‐standing questions about forest trees.

## 1 THE GLOBAL IMPORTANCE OF FORESTS

Just over 30% of the earth's land surface is covered by forests (FAO, 2015). Since the beginning of human civilization, the forest cover has been globally reduced by nearly half, with forests felled over previous centuries in the industrialized world and most current deforestation occurring in the tropics (Crowther et al., 2015; FAO, 2015). Trees are the ecosystem architects of forests, providing habitat for biodiversity—around 80% of terrestrial species globally are forest dwelling. Forest trees also provide human communities with food, fiber, and fuel. They play a critical role in the global carbon cycle and, along with other terrestrial vegetation, have sequestered more than a quarter of the additional carbon dioxide released by humans during the Anthropocene period (Le Quéré et al., 2018). Trees are the most efficient and cost‐effective carbon capture and storage technology available (Lewis, Wheeler, Mitchard, & Koch, 2019). Bastin, Finegold, Garcia, Mollicone, et al. (2019) have highlighted the potential of global tree restoration that could address the global carbon problem; however, simply planting trees on all suitable land is a solution fraught with shortcomings (Bastin, Finegold, Garcia, Gellie, et al., 2019; Lewis, Mitchard, Prentice, Maslin, & Poulter, 2019). Deforestation and forest degradation are responsible for approximately 20% of carbon emissions (Pearson, Brown, Murray, & Sidman, 2017). Tree mortality due to drought, wildfire, insects, and diseases is turning some forests from carbon sinks to carbon sources through a dangerous positive feedback cycle (Allen, Breshears, & McDowell, 2015; Kurz et al., 2008). While tree planting alone will not solve the climate crisis, a better understanding of the genetics and genomics of forest trees could increase the success of reforestation and conservation initiatives, and inform projections of the capacity of tree populations to adapt to new environmental challenges.

## 2 A BRIEF HISTORY OF FOREST GENETICS

*“Trees have never been the ideal subject to study’’* (Aitken & Bemmels, 2015). Forest tree species are largely undomesticated. They generally exhibit high levels of genetic diversity resulting from large effective population sizes, local adaptation, and neutral evolutionary processes across heterogeneous environments (Hamrick, 1989). Most are outbreeding, with long lifespans and high levels of gene flow among populations (Kremer & Le Corre, 2012; Savolainen, Pyhajarvi, & Knürr, 2007). For over 200 years, forest biologists have used provenance trials to select seed sources for planting important commercial species and for assessing the distance a seed can be moved without compromising local adaptation under the assumption of stable climates (Aitken & Bemmels, 2015; Langlet, 1971). Operational tree breeding programs and related infrastructure (seed and seedling production) have been running since the mid‐1900s.

With the advent of molecular biology techniques, some research programs in the 1990s and early 2000s targeted QTL mapping in pedigreed populations to facilitate marker‐aided selection for growth, productivity, and wood attributes. These early investigations optimistically sought to identify one or a few major genes underlying traits of interest. It has since become clear that most economically or environmentally relevant traits are highly polygenic (Manolio et al., 2009). More recently, as sequencing costs have dropped, forest genetics has shifted from searching for single genes having a large effect, or testing smaller sets of candidate genes, to genome‐wide or exome‐wide scans to address pressing issues related to fiber production, climate adaptation, and resistance to biotic and abiotic stresses. Extensive genomic resources have now been developed for a number of economically important species, including reference genomes for black cottonwood (*Populus trichocarpa*), Eucalypts (*Eucalyptus grandis*), loblolly pine (*Pinus taeda*), Norway spruce (*Picea abies*), pedunculate oak (*Quercus robur*), and others (see Tree Genes database https://treegenesdb.org/).

In this issue, Pyhäjärvi, Kujala, and Savolainen (2019) provide an illustration of the extent to which genomic tools have advanced the field of forest genetics in Scots pine (*Pinus sylvestris*). Scots pine has one of the widest distributions of any tree species, was one of the earliest studied species, and remains one of the most studied. The first recorded provenance trial for any species was established in 1745, when seed from Scots pine trees from different European provenances was collected by and planted on the estate of H.L. Duhamel du Monceau in France (Langlet, 1971). A long tradition of provenance trials ensued. Subsequent studies characterized a close relationship between bud phenology and cold hardiness relative to provenance climate, a largely outcrossing mating system, and high inbreeding depression typical of many widespread conifers. Although the 22 gigabase Scots pine genome has not yet been sequenced and assembled, genomic approaches have nonetheless rapidly expanded knowledge of population differentiation as well as genomic diversity, evolution, local adaptation, and the architecture of traits. While the census size across the species’ range is in the billions of trees, the silent nucleotide diversity and mutation estimates yield an effective population size of tens to hundreds of thousands. This may be due to historical demographics, variation in fecundity, effects of linked selection, or a lower actual mutation rate than previously estimated. An excess of rare variants suggests an ancient bottleneck may have played a role. Scots pine, like many widespread conifers, has a very weak neutral population structure, but strong population differentiation for phenotypic traits, especially growth and bud phenology, along a climatic gradient from north to south. Facilitated by relatively large effective population sizes, populations have adapted to local climates during recolonization since the last glacial maximum. Since the neutral population structure also follows a north–south pattern, controlling for structure in genotype–environment or genotype–phenotype association studies remains challenging. Nonetheless, small effect genomic markers related to the timing of bud burst have been identified. Surprisingly, few markers have shown clinal patterns of allele frequencies across the species range, and different markers are associated with phenotypes in northern versus central populations. This speaks to a need to consider a broader range of methods for better understanding the covariance of alleles at different loci across complex environmental gradients (Kremer & Le Corre, 2012.

## 3 INSECT AND DISEASE RESISTANCE AND EARLY DETECTION

Most early studies of forest genetics focussed largely on traits of economic importance, primarily growth and wood quality. There is now an urgent need to study pest resistance as the impacts of non‐native insects and diseases grow, and as some native pest species have increased their ranges and impacts due to climate change. This issue features studies using genomic tools to: (a) understand the genomic architecture of insect and disease resistance or tolerance; (b) select for trees that can survive and thrive in the presence of these pests; and (c) detect invasive pests and pathogens.

The American chestnut (*Castanea dentata*) provides a dramatic example of what can happen when a dominant tree species collapses due to an introduced pathogen, and the role genetic resistance might play in its recovery. Westbrook et al. (2019) tell the fascinating story in this issue of the American chestnut and the different approaches taken to develop resistant trees. Since the beginning of the twentieth century, when it was introduced to North America, the chestnut blight fungus (*Cryphonectria parasitica*) has killed more than 4.2 billion American chestnut trees. Breeding programs that generated interspecific hybrids between Asian *Castanea* species and American chestnut were initiated in the USA in the 1920s but were abandoned in the 1960s. More recently, the American Chestnut Foundation (TACF) has implemented a breeding program that incorporates the blight resistance of Chinese chestnut (*Castenea mollissima*). Based on the hypothesis that blight resistance is conferred by only a few major loci, *C. mollissima* × *C. dentata* F_1_ were backcrossed to *C. dentata* over three generations. The resulting third‐generation backcross progeny were then intercrossed, resulting in 44,000 BC3F2 trees between 2002 and 2018. Blight resistance assessment following pathogen inoculation identified 7,600 BC3F2 trees as potential parents for the next round of crosses that will generate BC3F3 restoration populations. However, these BC3F2 trees must be progeny tested in order to cull all but 1% of the top potential parents. Since the evaluation of thousands of progeny is practically impossible, Westbrook et al. (2019) developed genomic prediction models for blight resistance and coupled these with estimates of species ancestry. They found that genomic prediction was more accurate than prediction based on pedigree alone, which means that the costly and time‐consuming inoculation of all BC3F3 progeny can be avoided. They also found that there was a trade‐off between blight resistance and the proportion of the genome inherited from *C. dentata*, and that resistance rather follows a polygenic inheritance pattern. As *C. mollissina* is a small‐statured tree, genotypes with low *C. dentata* ancestry are unlikely to become the forest giants that were once common. All this prompted TACF to consider two options to develop resistant trees: (a) inclusion of additional *C. mollissina* sources of resistance using a marker‐assisted introgression system and (b) the use of established transgenic technology to create resistant *C. dentata* (Newhouse et al., 2014).

While climate change alone is presenting significant challenges to the persistence and fitness of local tree populations, the interaction between a changing climate and the distribution and abundance of insect pests will be a complicating factor. This issue is perhaps nowhere better illustrated than in the mountain pine beetle (MPB; *Dendroctonus ponderosae*) system of western North America. Over the past two decades, the abundance of this native pest of pines increased exponentially, due in most part to warmer winter temperatures that are no longer sufficient to control populations (Safranyik et al. 2010). The MPB has caused substantial mortality over tens of millions of hectares of lodgepole pine (*Pinus contorta*) and in areas of sympatry with jack pine (*Pinus banksiana*) in western Canada. This has had profound ecological and economic consequences, as these species are dominant components of many forest ecosystems. There is substantial interest in breeding for MPB resistance to restore these areas. Cullingham et al. (2019) used a large population of the lodgepole‐jack pine species complex to test 17 previously identified candidate loci for associations with MPB resistance. The authors apply a variety of selection tests coupled with transcriptional profiling during the MPB attack and successfully identified two candidate loci that were consistently related to resistance across selection tests and upregulated in response to attack. Moreover, one of these loci also had a significant association with MPB resistance in lodgepole pine. This study shows that resistance loci segregate in lodgepole pine, and that future work at the genome‐wide scale may enable the development of marker‐aided selection or other genomic selection tools for breeding programs for these species.

Spruce budworm (*Choristoneura fumiferana*) is also a native insect herbivore with major economic repercussions, as it can kill 50% or more of its balsam fir (*Abies balsamea*) and spruce (*Picea* sp.) hosts during epidemic outbreaks. For this issue, Parent et al. (2019) review different lines of evidence supporting the important role of a recently identified chemical defense mechanism against this insect in white spruce (*Picea glauca*)*.* Accumulation of the hydroxyacetophenones, piceol, pungenol, and picein, correlates with the expression of *Pgβglu‐1*, and greater accumulation is associated with less defoliation by the spruce budworm. Resistant trees in field trials have higher levels of piceol, pungenol, picein, and *Pgβglu‐1* expression, and these chemical defense traits are generally highly heritable. There do not appear to be substantial trade‐offs between growth and accumulation of these defenses, making them good targets for selection in breeding programs. Biosynthesis of these compounds varies seasonally and ontogenetically. A genome‐wide association study with 4,767 SNPs identified 3–9 genes that explained 27–43% of the variation in piceol, pungenol, picein, and *Pgβglu‐1* expression levels. Parent et al. (2019) also present results of a spruce budworm feeding study showing hydroxyacetophenones reduced pupal mass, delayed development, and decreased the survival of larvae, providing additional evidence of the defensive role of these chemicals. Collectively, the evidence presented makes a convincing case for selection on these chemical defenses in spruce breeding programs.

Following major investments in the development of genomic resources, genomic selection for growth, wood traits, and pest resistance in breeding programmes were among the first genomic applications in forestry (e.g., Resende et al., 2012). The primary advantage of genomic selection over traditional progeny testing and selection is to shorten the length of a breeding cycle, which for trees can be as long as 30 years. One challenge for selection is to consider multiple phenotypic traits. In this issue, Lenz et al. (2019) compared single‐ and multi‐trait genomic selection approaches in Norway spruce (*Picea abies*) that target tree productivity, wood quality, and insect resistance. This species was introduced to Eastern Canada in the early 1900s for plantation forestry and has proved to be very productive, except that it is susceptible to the native white pine weevil (*Pissodes strobi*). Although Norway spruce has not co‐evolved with this insect, genetic variation for resistance was fortuitously observed among Norway spruce seed sources. Lenz et al. (2019) demonstrate how multi‐trait genomic selection models can help breeders resolve complex issues such as breeding for multiple criteria. Encouragingly, the case of Norway spruce shows that it is possible to breed simultaneously for insect resistance and productivity.

To minimize the risk of new pest invasions with catastrophic ecological consequences like chestnut blight on American chestnut, or more recently the emerald ash borer (*Agrilus planipennis*) on *Fraxinus* sp., biosurveillance systems are urgently needed. In this issue, Hamelin and Roe (2019) present a review of recent advances made in the development of genomic biosurveillance within a generalized framework that can be used to prevent or control exotic pests at an early stage of introduction to slow invasions. They illustrate how genomic tools already contribute to each of the critical stages in detection, from the taxonomic identification of specimens to determination of the origins of intercepted specimens and their invasion routes. At the same time, to help prioritize the actions of regulatory agencies, they provide useful advice on how predictive models for risk assessment could benefit from genomic approaches. Genomics will likely transform the management of invasive species, as is the case now for human epidemics that are monitored in real time. To achieve this, mobile detection technologies are needed for field sample analysis in remote areas, and several of these are either already available or in development. Engaging end‐users in tool development will increase the likelihood of their adoption.

## 4 ADAPTATION TO CHANGING CLIMATES

To keep pace with climate change, forest managers are seeking decision‐making tools and guidance for restoration and reforestation decisions. Some of the papers in this issue highlight how population genomic approaches may be able to provide an understanding of the adaptive capacity of tree populations and the challenges that warmer climates will bring. Many tree species have both high levels of genetic diversity and considerable phenotypic plasticity that have allowed them to survive past environmental variability. However, we need better knowledge of the extent of current local adaptation of many tree species to climate, and new methods for predicting the capacity to tolerate or adapt to new climates.

One expectation under climate change is that the climatic niche of individual species will shift outside current species ranges (Aitken, Yeaman, Holliday, Wang, & Curtis‐McLane, 2008; Parmesan, 2006). Traditionally, long‐term field provenance trials have provided high‐quality information on population variation in growth and adaptation to climate for some economically important species. However, very few of these trials have been planted outside of current species distributions and the majority of tree species have no such trials (e.g., Marris, 2009; McLane & Aitken, 2012). Short‐term seedling common gardens and genomic approaches are alternatives for rapidly identifying the environmental factors driving phenotype–environment associations, and identifying genotype–phenotype and genotype–environment associations (Alberto et al., 2013; Sork et al., 2013).

There is a rapidly increasing number of studies in forestry that are evaluating the potential of genomics to gauge adaptive variance in comparison with phenotypic data obtained from common gardens. For example, Mahoney et al. (2019) evaluate the performance of genomic data compared with phenotypes measured in a short‐term seedling common garden (bud phenology, cold hardiness, and growth) and long‐term provenance trials (20 year‐height) to determine patterns of adaptive variation and climatic drivers of selection. These data were combined with genotypes for > 32,000 candidate SNPs and climatic data for 281 populations of lodgepole pine (*Pinus contorta*), a commercially and ecologically important species. They found that markers associated with seedling phenotypes in a common garden explained adaptive variation better than the genomic dataset or climatic data alone. They also show that genotype–environment association (GEA) analyses could be used as a surrogate to identify climatic drivers when phenotypic data (e.g., 20 year‐height in a provenance trial) are not available, an approach that may help guide species management without long‐term trials. Most climate‐related SNPs were polymorphic in most populations, suggesting considerable standing variation for adaptation to new climates.

One of the key expected outcomes of applied ecological genomics is the prediction of the evolutionary potential of local populations in the context of climate change. While there has been much discussion in the field as to the potential of predicting allele frequency shifts for adaptive loci that will be required to track shifting climates, there are few actual results. For this special issue, Ingvarsson and Bernhardsson (2019) address this question using *Populus tremula* (European aspen), a widespread species with a long history of genetic, genomic, and phenotypic studies. The authors completed whole‐genome sequencing of 94 individuals originating from across 10 degrees of latitude in Sweden. These data were combined with genotype–environment analyses (LFMM), and modeling of the environmental context of adaptive alleles (GDM), to estimate “genetic offsets” (Fitzpatrick & Keller, 2015) that describe expected maladaptation when an individual of a particular allelic composition is transferred from its origin to a novel environment. Within a relatively short period (~50 years), the northern populations in particular are expected to require large genetic adjustments to remain adapted to their local climate. By contrast, southern populations will experience smaller genetic offsets when experiencing to future climates. This is consistent with the disproportionate effects of climate change in the sub‐arctic (IPCC, 2018).

Although much of the forest genomics literature focuses on economically important species, the power of population genomics is also being applied to understand neutral and adaptive processes in noncommercial species of ecological value. Mayol et al. (2019) provide one such example for English yew (*Taxus baccata*) in this issue. While widely distributed across Europe and not presently threatened, English yew tends toward small population sizes that may have limited adaptive potential, making it vulnerable to climate change. In this study, the authors leverage an existing common garden of 26 yew populations from the Iberian Peninsula to understand phenotypic adaptation, and found evidence for adaptive divergence in growth and phenology that is correlated with temperature variation among the provenances. Incorporating additional samples from across the natural species range, the authors then used sequence capture to genotype ~1,200 genes and coupled these data with tests of environmental selection and modeling of the demographic history of the species. Their results suggest that, unlike most temperate and boreal tree species, European yew has experienced demographic decline throughout the late Quaternary, which is more pronounced in some populations than in others. The most novel aspect of the study was the use of pathway analysis to assess the joint effect of SNPs in different biological categories. This approach revealed that, among others, the flavonoid biosynthesis pathway has experienced diversifying selection across the yew range, which reflects their functional relevance in oxidative stress or membrane stabilization in cold temperatures (Schulz, Tohge, Zuther, Fernie, & Hincha, 2016). As population genomics moves from descriptions of univariate SNP–environment and SNP–phenotype relationships, which lack power (Kremer & Le Corre, 2012), toward a view of adaptation as a polygenic process encompassing many loci of vanishingly small effect (Boyle, Yang, & Pritchard, 2017), this pathway approach is likely to see wider use to understand how genotypes maps to phenotypes.

Assisted gene flow is being considered, or in some cases has already been implemented, as a forest management option to address climate change, as it can introduce or increase the frequency of pre‐adapted genotypes for new climates (Aitken & Whitlock, 2013). If assisted gene flow is used, it should rely on the best available information about local adaptation for a given species. Genomic data could enrich conservation strategies by providing robust estimates of among‐population adaptive variation (Funk, Forester, Converse, Darst, & Morey, 2019). For less‐studied species, current indices in use (e.g., Shapley Index) are often based on neutral diversity estimates that do not reflect adaptive variation. In their study presented in this issue, Borrell, Zohren, Nichols, and Buggs (2019) report the case of montane dwarf birch (*Betula nana*), a tree species that is experiencing an accelerated decline in the UK due to anthropogenic activities including climate change. Although conservation and restoration programs are underway, the authors suspect that the future adaptive capacity of *B. nana* may not be adequate. Before designing an assisted gene flow strategy for *B. nana*, the authors used genotype‐by‐environment association (GEA) and environmental niche modeling approaches to identify maladapted populations, that is, those that show allele frequency deviation under current and future environmental conditions (c‐RONA and f‐RONA). A consensus ranking between the Shapley Index and c‐RONA that permits the maximization of the adaptive variation of the populations surveyed was developed. Borrell et al. (2019) discuss the potential of this well‐informed assisted gene flow framework and the limits of such an approach.

Assessing a species adaptive capacity to provide relevant information for vulnerability assessments remains challenging, especially for nonmodel species like North American *Populus deltoides* W. Bartram ex Marshall. In this issue, Godbout, Gros‐Louis, Lamothe, and Isabel (2019) examine how genomic diversity in this foundation species was shaped by its environment (climate, soil, and biotic interactions) to gauge its adaptive capacity. They used two complementary approaches to get a full portrait of *P. deltoides* genetic diversity at both the species (~1,000 individuals, 90 populations) and whole genome levels. The first step, based on a modest number of chloroplast and nuclear SNP gene‐based markers, allowed the detection of a strong geographic structure related to three different existing *P. deltoides* lineages and evidence of uneven gene flow among the groups. These findings were confirmed by the use of a sequence capture approach using DNA pools, representative of each detected lineage. With a modest budget, this study has enabled the authors to make soft recommendations to forest managers to maintain the adaptive variation of *P. deltoides*.

## 5 EVOLUTIONARY IMPORTANCE OF HYBRIDIZATION IN TREE SPECIES

Many tree species hybridize with congeners, which potentially contributes to local adaptation in transitional environments (Bawa & Holliday, 2016). For example, recent studies in the *Populus trichocarpa* × *P. balsamifera* hybrid zone in western North America suggest that directional introgression from *P. balsamifera*, which occupies cold continental environments, into *P. trichocarpa*, which occupies warm coastal environments, enables the latter to occupy colder habitats than is otherwise not typical of the species range (Suarez‐Gonzalez, Hefer, Lexer, Douglas, & Cronk, 2018). Menon et al. (2019) address a similar question in this issue for the hybridization of western conifers *Pinus strobiformis* and *P. flexilis*. The former occupies a more southern range extending from Arizona (USA) into Mexico, while the latter is more northerly, mostly distributed in the western intermountain region of the USA. They sampled >300 trees from 21 populations, of which 16 were within the putative hybrid zone in southern Arizona and northern Mexico. They also collected morphological data that delineates the species, sequenced each tree with a double‐digest RADseq and combined these data with individual‐based simulations and genomic cline analyses. Their results suggest that introgression is facilitating the northward movement of the *Pinus strobiformis* range through directional introgression of adaptive alleles from *P. flexilis*. The authors also show that the hybrid zone between these two species may be moving northward, as evidenced by a lack of concordance between morphological and genomic cline center estimates. While hybridization is sometimes viewed as degrading biodiversity, particularly for threatened species, introgression between forest tree species is a natural feature of their evolutionary trajectory, providing intermediate phenotypes within ecotones as well as the bi‐directional transfer of adaptive variation. Menon et al. (2019) provide one of the few examples of this transfer at the genomic level in a tree species complex, with important implications for adaptation that are not often incorporated into predictive frameworks of species responses under climate change.

## 6 UNDERSTANDING GENOME EVOLUTION AND GENERATING GENOMIC TOOLS

The size and complexity of tree genomes, especially those of conifers, has until recently hampered the attainment of high‐quality reference genomes. The first tree genome to be sequenced was *Populus trichocarpa* in 2006 (Tuskan et al., 2006), and the first conifer was *Picea abies* (Nystedt et al., 2013). Of the ~200 unique plant reference genomes published, 52 are tree species (Wegrzyn et al., 2019). With the advent of new genomic, bioinformatic and precision phenotyping tools, the situation, although not perfect, has significantly improved.

Gymnosperms and angiosperms diverged around 300 Mya. There are approximately 60,000 tree species in the world, and the vast majority of these are angiosperms. Only 588 tree species are conifers and, adding *Ginkgo biloba*, 589 are gymnosperms. Angiosperm genomes are much better understood than those of conifers, largely due to the enormous size and high repetitive element content of conifer genomes, which make assembly and bioinformatics challenging (De La Torre et al., 2014). Understanding conifer biology for divergent traits such as reproduction and wood anatomy is important, as they dominate approximately one third of forests globally. Advances in DNA sequencing technologies have led to significant breakthroughs in organisms with large genomes such as gymnosperms. The genomic resources that now exist have allowed De La Torre et al. (2019) to review the major differences between gymnosperm and angiosperm genomes and allowed them to conduct a comparative gene family analysis. Angiosperm evolution has involved considerable whole‐genome duplication events (Leebens‐Mack et al., 2019). These events are rare in gymnosperms, and thus, high levels of macrosynteny and collinearity have persisted among conifer species despite their divergence more than 140 million years ago (Pavy et al., 2017). Chromosomal rearrangements are also rare, and mutation rates are slow compared with angiosperms. However, gymnosperm genomes are large due to the accumulation of highly repetitive elements, especially long‐terminal repeat retrotransposons, which comprise 75% or more of their genomes. How the genome size of gymnosperms may have influenced the adaptation process is still not well studied (Mei, Stetter, Gates & Ross‐Ibarra,^2018^). As a starting point, coding regions are used to compare rates of adaptive evolution between gymnosperms and angiosperms. While gymnosperms have lower rates of neutral evolution than angiosperms, they have higher rates of nonsynonymous substitutions (De la Torre, Li, Peer, & Ingvarsson, 2017). Despite differences in genome evolution, gymnosperms and angiosperms have similar numbers of genes and gene families, with some exceptions. Compared to angiosperms, gymnosperms have had greater expansion of gene families involved in defense responses (including terpenoids), cold and drought tolerance, and lignin and cellulose biosynthesis. De La Torre et al. (2019) also identify several gene families that may have potential for industrial or pharmacological development in an emerging bioeconomy**.**

Genomics alone cannot answer all questions concerning how populations adapt to their environment. Studying adaptation to biotic and abiotic stresses across environmental gradients along tree lifespans also requires the evaluation of phenotypes for large populations. The phenomics era, with tools such as UAVs that fly over test sites with hyperspectral sensors that generate leaf spectral indices as proxies to monitor phenology, responses to drought stress or pest invasions, is already here (e.g., Calderon, Navas‐Cortés, & Zarco‐Tejada, 2015). Precision phenotyping opens up new research avenues that could help tackle specific questions like resistance to biotic and abiotic stress, for example. Despite the availability of low‐cost high‐throughput technologies to generate genomic and phenotypic data, forest biologists working with large populations of long‐lived forest trees are constantly facing new challenges. As reported by Wegrzyn et al. (2019), the current state of data integration for nonmodel species is far from what exists for model species. To avoid reinventing the wheel, and to minimize storage redundancy, optimize query data and keep pace with technological development, nonmodels would greatly benefit from the development of cyberinfrastructures that support all aspects of the data life cycle, from acquisition to storage, integration, and visualization. However, implementing and sustaining cyberinfrastructures requires the significant improvement of practices in four main areas: data standards, ontologies, analytic workflows, and integrated databases. Wegrzyn et al. (2019) describe these, along with examples of the best practices in use by the genomics community. One of the most critical pieces is the determination of data standards. Initiatives such as FAIR (Findability, Accessible, Interoperable, Reusable) sharing should be mimicked and encouraged by journal and funding agencies. The implementation of cyberinfrastructures based on best practices will benefit all research communities.

## 7 CONCLUSION

The long‐term maintenance of forest health is of fundamental importance to global ecosystem functions and processes in general, and in the fight against climate change in particular. Forest trees, the ecosystem architects of forests, are challenged throughout their lifespan by a myriad of abiotic and biotic stresses, and their maladaptation to climate change may increase their susceptibility to native and exotic pests or other natural disturbances (Allen et al., 2010). As demonstrated in this special issue, genomics is now making it possible to rapidly improve knowledge and develop tools to inform forest management and conservation decisions, and to accelerate breeding programs, to face the challenges of climate change, even in nonmodel species. To ensure the acceptance of these new tools and approaches by the various stakeholders, ranging from the public to decision makers, economic and ecological costs and benefits, as well as social license for applications, must be understood. These particular aspects should not be underestimated, especially when difficult choices need to be made.

## CONFLICT OF INTEREST

None declared.

## AUTHOR CONTRIBUTIONS

NI, JH, and SA conceived the project and wrote the manuscript.
